# Supplementary material for: Network-based approach to prediction and population-based validation of in silico drug repurposing
Source: Nat Commun. 2018 Jul 12;9:2691. doi: 10.1038/s41467-018-05116-5 (PMC6043492; doi:10.1038/s41467-018-05116-5)
Supplement: Supplementary file 1 — Supplementary Information [file 41467_2018_5116_MOESM1_ESM.pdf]

SUPPLEMENTARY INFORMATION FOR

Network-based Approach to Prediction and Population-based Validation of *in silico* Drug Repurposing

Cheng et al., *Nature Communications* 2018

*Corresponding author: Email: jloscalzo@rics.bwh.harvard.edu (J.L.)

The PDF file includes:

Supplementary Figure 1. Schematic illustration of the integrated, network-based, systems pharmacology approach.

Supplementary Figure 2. The receiver operating characteristic curves of network proximity quantifying the interplay between disease genes and drug targets on the human interactome for identifying well-known drug-cardiovascular (CV) associations between 177 FDA-approved CV drugs and 23 types of CV events.

Supplementary Figure 3. The receiver operating characteristic curves of the closest distance-based network proximity score compared to three different network distance-based measures.

Supplementary Figure 4. Hazard ratios and 95% confidence intervals for the associations of interest - Truven Marketscan database.

Supplementary Figure 5. Hazard ratios and 95% confidence intervals for the associations of interest - Optum Clinformatics database.

Supplementary Figure 6. Mechanism-of-action for lithium's causal effect on stroke by network analysis and *in vitro* assay.

Supplementary Figure 7. Uncropped images for Figure 4c Western blots.

Supplementary Table 1. The statistics of disease-associated genes for 23 cardiovascular outcomes.

Supplementary Table 2. Patient characteristics, Carbamazepine vs. Levetiracetam, 1:1 propensity score-matched. (.PDF)

Supplementary Table 3. Patient characteristics, Mesalamine vs. Azathioprine/6-MP, 1:1 propensity score-matched. (.PDF)

Supplementary Table 4. Patient characteristics, Lithium vs. Lamotrigine, 1:1 propensity score-matched. (.PDF)

Supplementary Table 5. Patient characteristics, Hydroxychloroquine vs. Leflunomide, 1:1 propensity score-matched. (.PDF)

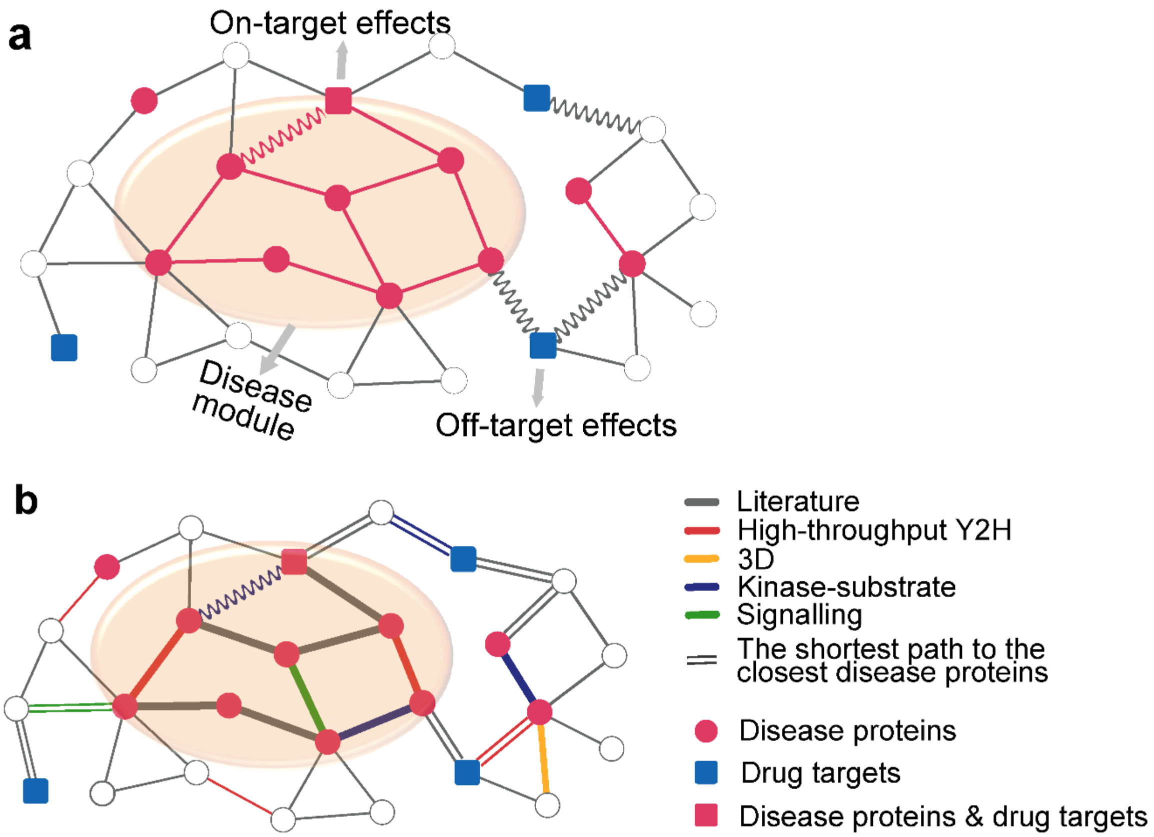

Supplementary Figure 1. Schematic illustration of the integrated, network-based, systems pharmacology approach. (**a**) The network proximity approach asserts that for a drug to be effective or cause cardiac side effects, it must target multiple proteins within or in the immediate vicinity of the corresponding cardiovascular disease (CVD) module. 'Spring' lines show the interactions between drug primary targets and disease module. (**b**) Network-based drug-disease proximity is quantified by determining the shortest paths between drug targets and disease proteins in the human protein-protein interactome (*cf.* Methods). Five types of human experimental protein-protein interactions are used in this study (*cf.* Methods).

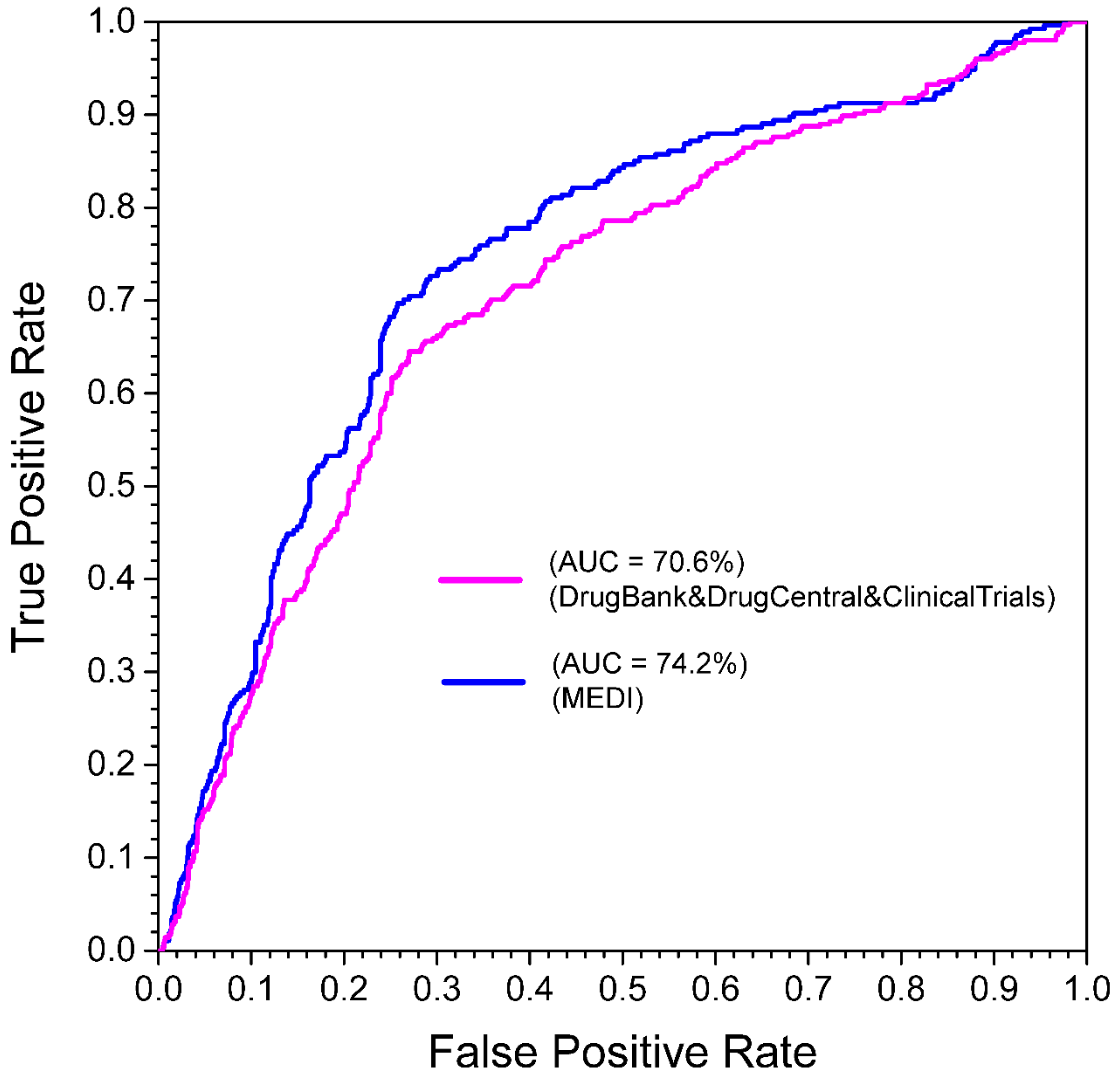

Supplementary Figure 2. The receiver operating characteristic curves of network proximity quantifying the interplay between disease genes and drug targets on the human interactome identify well-known drug-cardiovascular (CV) associations between 177 FDA-approved CV drugs and 23 types of CV events. We collected the well-known drug indications from two types of data: (i) FDA-approved drug indications from DrugBank database[1] (v4.3) and DrugCentral[2] and clinically investigational drug indications from ClinicalTrials.gov, called DrugBank&DrugCentral&ClinicalTrials (pink curve); (ii) computable drug indications from four public mediation resources using natural language processing approaches[3], called MEDI (blue curve). AUC: the area under the receiver operating characteristic curves.

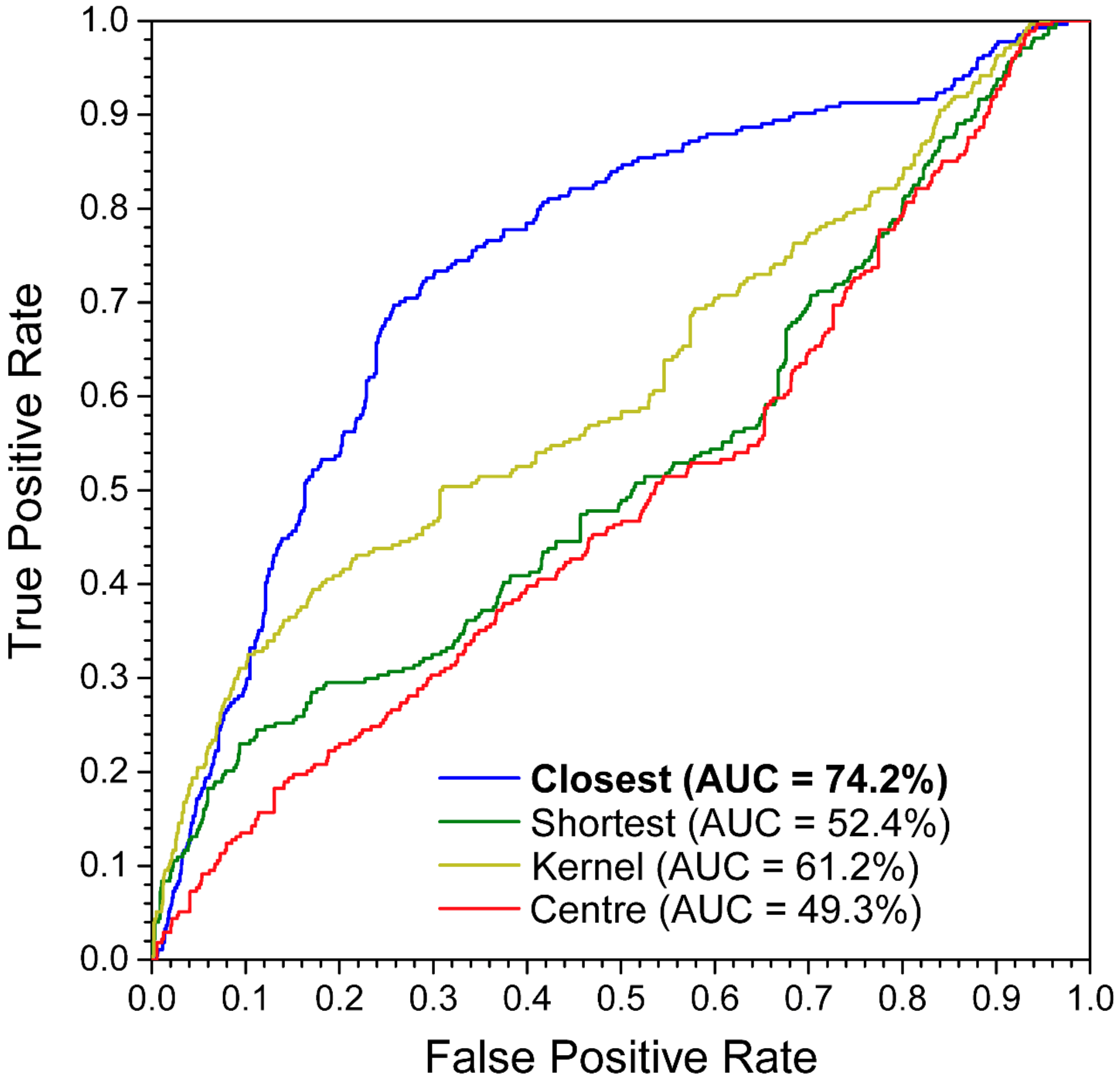

Supplementary Figure 3. The receiver operating characteristic curves of the closest distance-based network proximity score (Equation 1) compared to three different network distance-based measures: Shortest, kernel, and centre, as described in a previous study[4]. The area under the receiver operating characteristic curves (AUC) was evaluated based on the computable drug indications collected from four public mediation resources using natural language processing approaches[3].

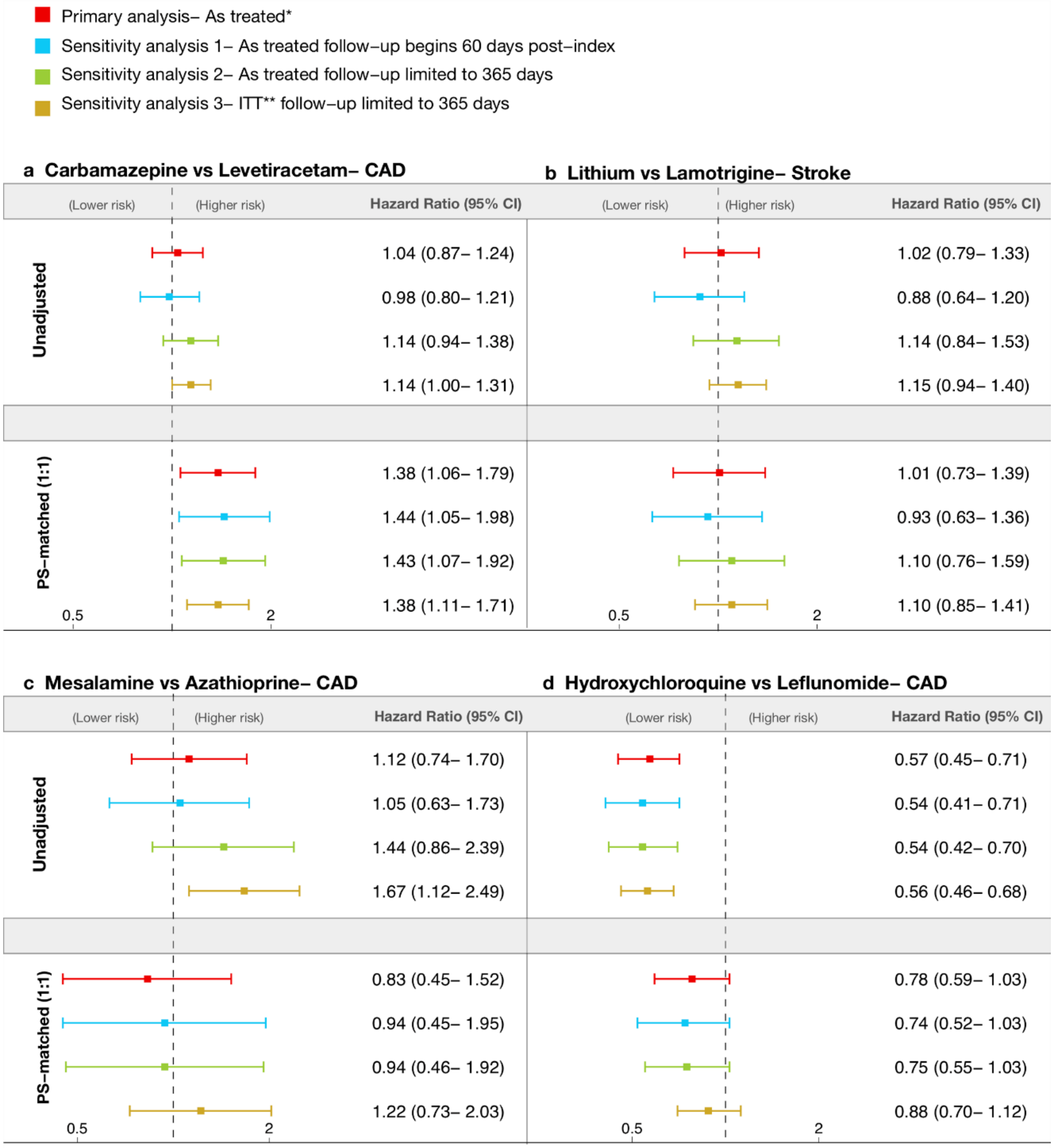

Supplementary Figure 4. Hazard ratios and 95% confidence intervals for the associations of interest- Truven Marketscan database. In the primary analysis approach (as treated), the follow-up was stopped upon discontinuation of the index medication. Follow-up assumptions were varied in three sensitivity analyses to: 1) exclude the first 60 days of follow-up to reduce unmeasured baseline confounding, 2) truncate the follow-up to 1-year to minimize time-varying confounding, and 3) continue the follow-up for 1-year regardless of treatment discontinuation under an intent-to-treat (ITT) principle. Propensity score (PS) matching accounted for >50 relevant patient characteristics; all analyses were conducted using DerSimonian and Laird random effects model with inverse variance weights. * In the ‘as-treated’ approach, the follow-up was stopped if patients either filled a prescription for a drug in the other exposure group or discontinued the index exposure. ** In the ITT analysis, patients were followed in their index exposure group regardless of treatment change or discontinuation for up to 365 days.

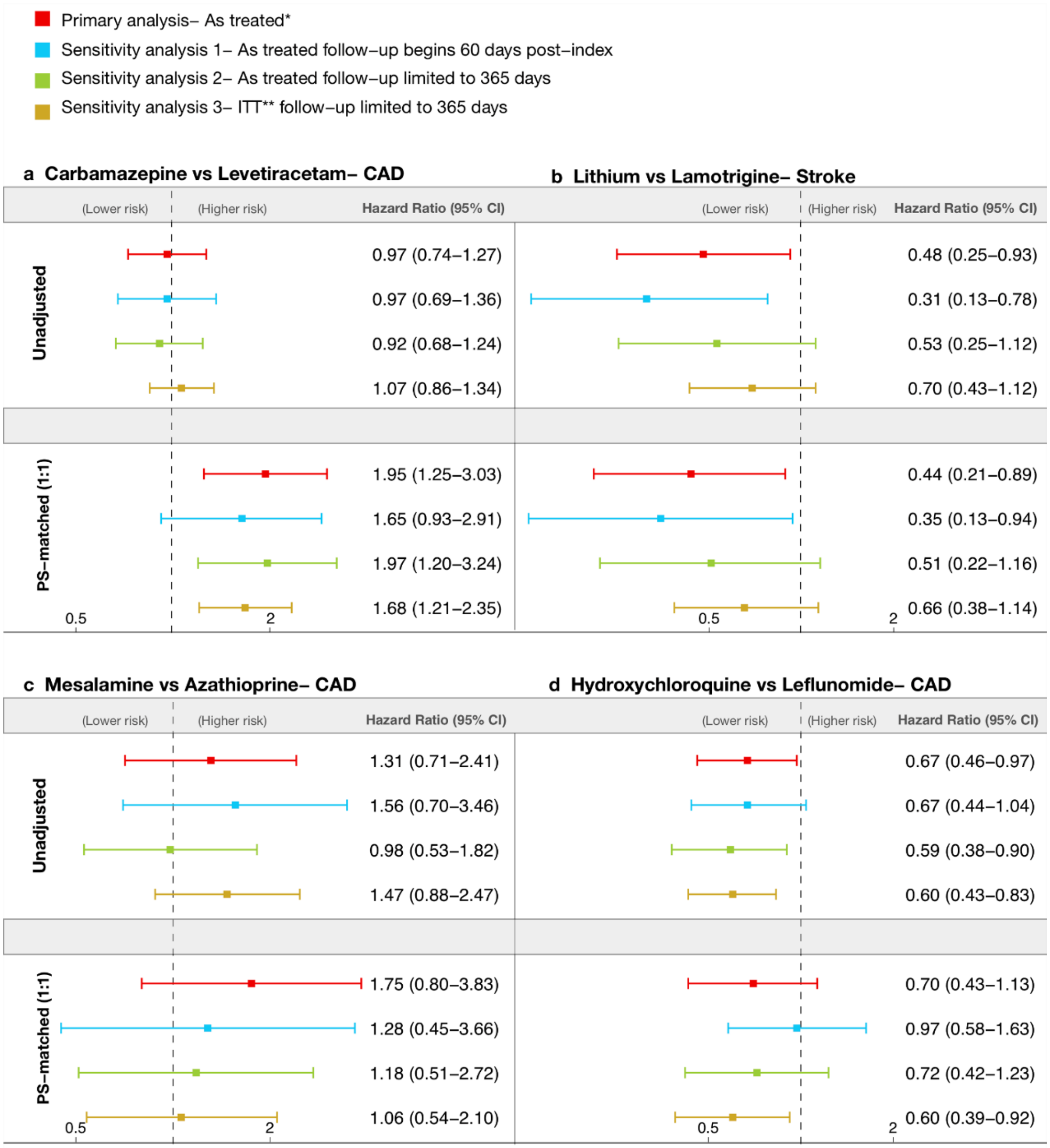

Supplementary Figure 5. Hazard ratios and 95% confidence intervals for the associations of interest- Optum Clinformatics database. In the primary analysis approach (as treated), the follow-up was stopped upon discontinuation of the index medication. Follow-up assumptions were varied in three sensitivity analyses to: 1) exclude the first 60 days of follow-up to reduce unmeasured baseline confounding, 2) truncate the follow-up to 1-year to minimize time-varying confounding, and 3) continue the follow-up for 1-year regardless of treatment discontinuation under an intent-to-treat (ITT) principle. Propensity score (PS) matching accounted for >50 relevant patient characteristics; all analyses were conducted using DerSimonian and Laird random effects model with inverse variance weights. * In the 'as-treated' approach, the follow-up was stopped if patients either filled a prescription for a drug in the other exposure group or discontinued the index exposure. ** In the ITT analysis, patients were followed in their index exposure group regardless of treatment change or discontinuation for up to 365 days.

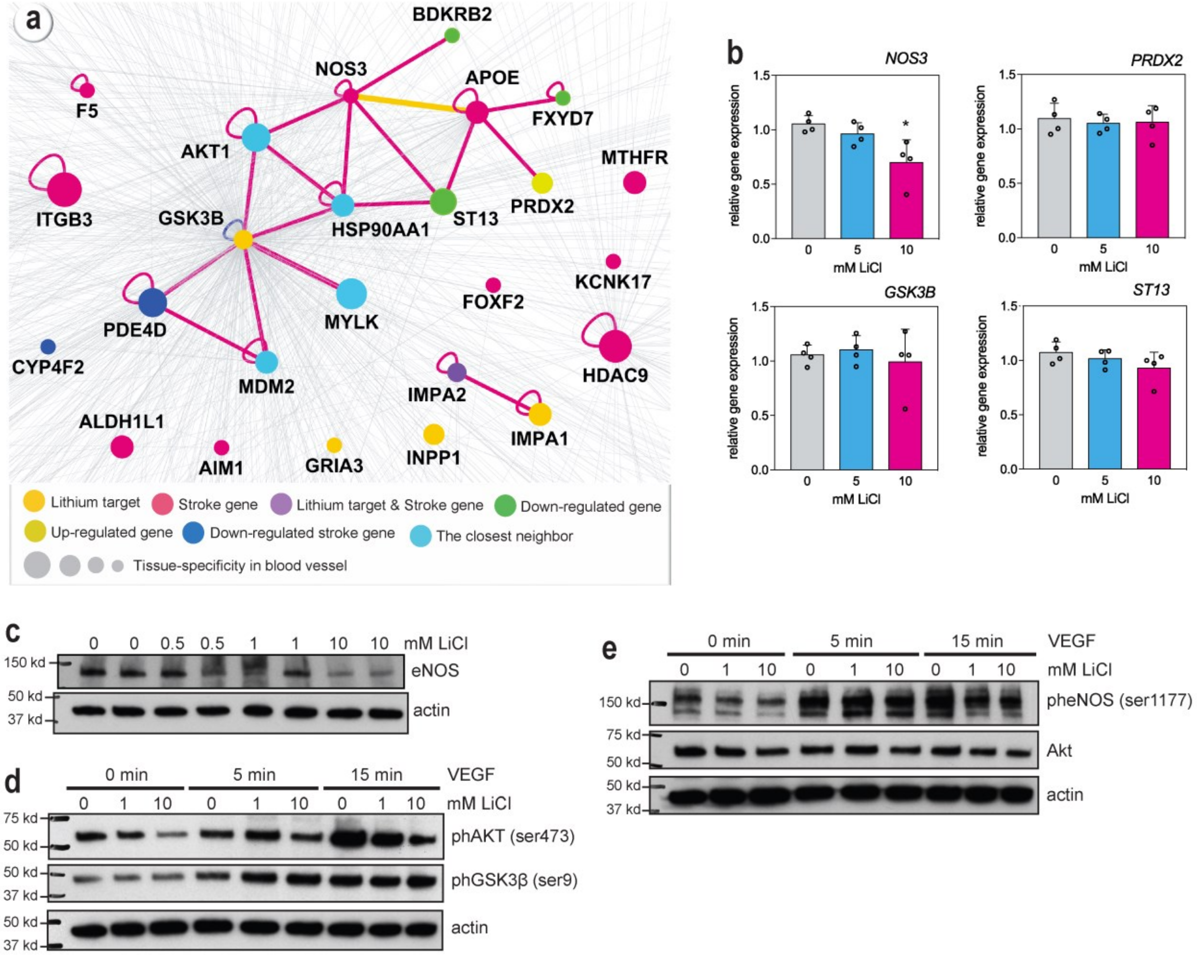

Supplementary Figure 6. Mechanism-of-action for lithium's causal effect on stroke by network analysis and *in vitro* assay. **a** A highlighted subnetwork shows the identified mechanism-of-action for lithium protective effect on stroke by network analysis. The node size scales show the blood vessel-specific expression level based on the Genotype-Tissue Expression data (see Methods). **b** Gene expression of *NOS3*, *PRDX2*, *GSK3B*, and *ST13,* normalized to that of ACTB (the beta- actin gene) after 24-hour exposure to lithium chloride (LiCl). Error bars indicate SEM; n=4. * p-value < 0.02; for trend (ANOVA). **c** Protein expression of eNOS after 7-day treatment with LiCl. **d** & **e** Cells were treated with various doses of LiCl in media with 0.1% FCS for 24 h, then stimulated with 50 ng/ml VEGF. **d** Phosphorylation level of GSK3β and Akt. **e** Phosphorylation level of eNOS. TaqMan Reagents from ThermoFisher used for Gene expression studies: *NOS3*, Hs01574659_m1; *GSK3B* Hs001047719_m1; PRDX2, Hs00853603_s1; ST13, Hs00832556_SH, and ACTB, Hs99999903_m1. Antibodies (Cell Signaling) used for Western Blotting: phAkt (ser473) #4060 (1:1000); Akt # 9272 (1:2000); phGSK3β (ser9) #5558 (1:2000); pheNOS (Ser1177) #9570 (1:1000); eNOS # 5880 (1:1000); β-actin #4970 (1:4000). Secondary antibodies used were: anti-rabbit HRP #7074 (1:2000) or anti-mouse HRP #7076 (1:2000).

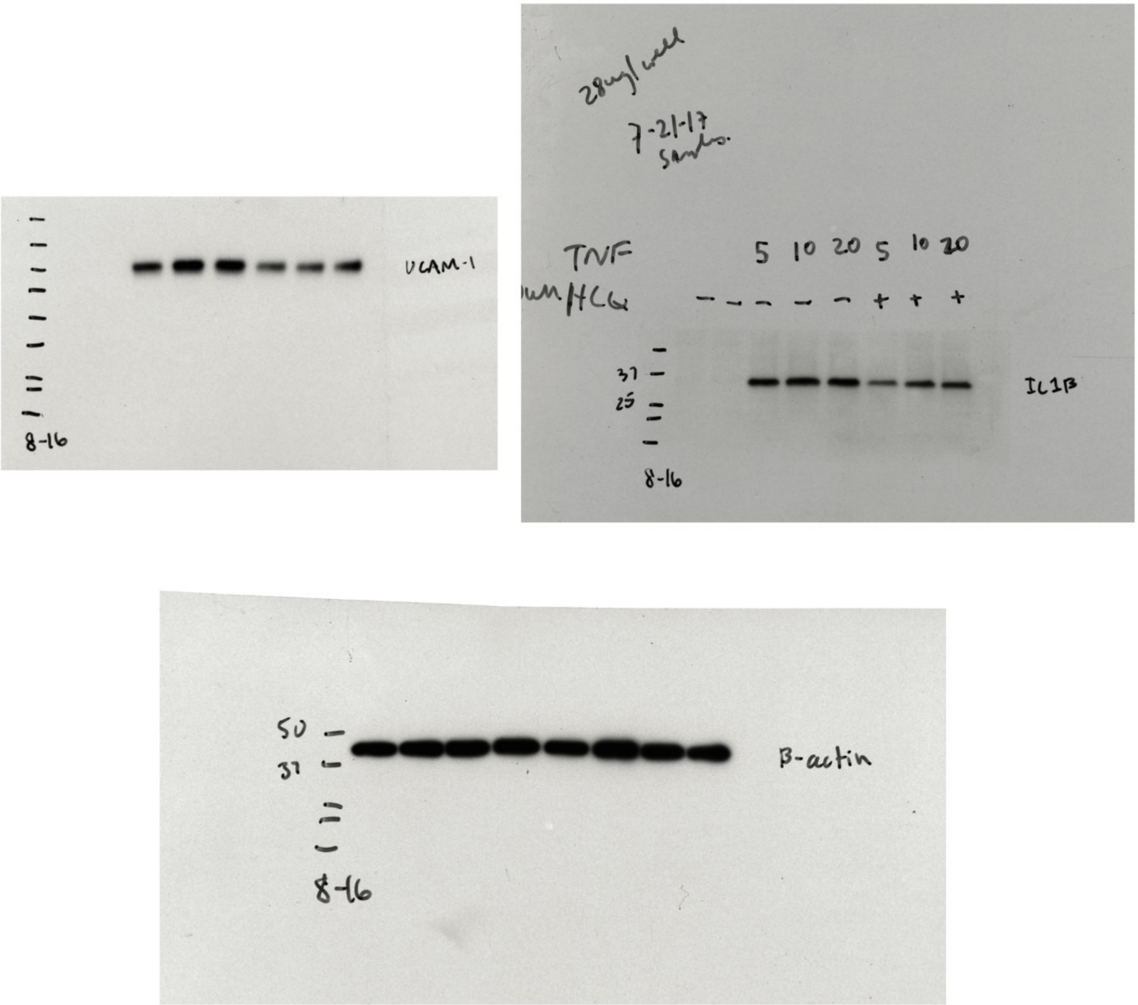

Supplementary Figure 7. Uncropped images for Figure 4c Western blots. Human aortic endothelial cells were pretreated with 10 μM hydroxychloroquine for one hour followed by exposure to 5, 10 or 20 ng/ml TNF-α. After 24 h, cells lysates were prepared. Samples were separated on polyacrylamide gels and transferred to PVDF membranes. VCAM-1 was detected by Western blotting using an sc-8304 antibody (Santa Cruz) at a 1:4000 dilution; IL-1β actin was detected using a 1:1000 dilution of antibody #12703 (Cell Signaling); and actin was detected using a 1:4000 dilution of antibody #4970 (Cell Signaling). A secondary anti-rabbit-HRP antibody (Cell Signaling, #7074) was used together with the ECL Western blotting detection reagents from GE Healthcare. Blots were exposed to X-ray film, and the Biorad ChemiDoc Touch Imaging system was used to generate images.

Supplementary Table 1. The statistics of disease-associated genes for 23 cardiovascular events.

Disease name	MeSH ID	Number of disease genes
Arrhythmia	D001145	335
Atherosclerosis	D050197	596
Cardiac congenital anomalies	C535853	95
Cardiomegaly	D006332	360
Cardiomyopathy	D009202	255
Cardiovascular abnormalities	D018376	176
Cardiovascular diseases	D002318	1702
Carotid artery diseases	D002340	152
Coronary artery disease	D003324	610
Coronary disease	D003327	781
Coronary restenosis	D023903	71
Coronary stenosis	D023921	109
Coronary thrombosis	D003328	29
Coronary vasospasm	D003329	21
Heart arrest	D006323	199
Heart block	D006327	15
Myocardial disease	D006331	876
Heart failure	D006333	266
Heart valve diseases	D006349	89
Hypertension	D006973	834
Hypertension, Pulmonary	D006976	55
Hypertension, Renal	D006977	30
Stroke	D020521	13

Supplementary Table 2. Patient characteristics, Carbamazepine vs. Levetiracetam, 1:1 propensity score-matched.

	Truven			Optum		
**Variable**	**Carbamazepine exposure**	**Levetiracetam exposure**	**Difference**	**Carbamazepine exposure**	**Levetiracetam exposure**	**Difference**
**Number of patients**	58,686	58,686	-	17,359	17,359	-
**Demographics**						
Age; mean (sd)	51.02 (17.72)	50.99 (17.19)	0.03 (-0.17, 0.23)	45.00 (14.96)	45.67 (15.04)	0.66 (0.35, 0.98)
Female; n (%)	33,748 (57.5%)	33,708 (57.4%)	0.1% (-0.5%, 0.6%)	9,870 (56.9%)	9,884 (56.9%)	0.1% (-1.0%, 1.1%)
**Mental health diagnoses**						
Epilepsy; n (%)	12,659 (21.6%)	13,044 (22.2%)	-0.7% (-1.1%, -0.2%)	6,220 (35.8%)	6,226 (35.9%)	0.0% (-1.0%, 1.0%)
Anxiety; n (%)	1,730 (2.9%)	1,751 (3.0%)	-0.0% (-0.2%, 0.2%)	678 (3.9%)	662 (3.8%)	-0.1% (-0.5%, 0.3%)
Depression; n (%)	2,323 (4.0%)	2,301 (3.9%)	0.0% (-0.2%, 0.3%)	954 (5.5%)	887 (5.1%)	-0.4% (-0.9%, 0.1%)
Sleep disorders; n (%)	3,202 (5.5%)	3,152 (5.4%)	0.1% (-0.2%, 0.3%)	1,371 (7.9%)	1,354 (7.8%)	-0.1% (-0.7%, 0.5%)
Bipolar disorder; n (%)	117 (0.2%)	147 (0.3%)	-0.1% (-0.1%, 0.0%)	47 (0.3%)	38 (0.2%)	-0.1% (-0.2%, 0.1%)
Drug or alcohol abuse; n (%)	2,902 (4.9%)	3,115 (5.3%)	-0.4% (-0.6%, -0.1%)	1,447 (8.3%)	1,275 (7.3%)	-1.0% (-1.6%, -0.4%)
Psychotic disorders; n (%)	1,940 (3.3%)	1,979 (3.4%)	-0.1% (-0.3%, 0.1%)	730 (4.2%)	693 (4.0%)	-0.2% (-0.6%, 0.2%)
Neuropathic pain; n (%)	7,493 (12.8%)	7,777 (13.3%)	-0.5% (-0.9%, -0.1%)	2,463 (14.2%)	2,435 (14.0%)	-0.2% (-0.9%, 0.6%)
Migraine; n (%)	3,667 (6.2%)	3,557 (6.1%)	0.2% (-0.1%, 0.5%)	1,751 (10.1%)	1,735 (10.0%)	-0.1% (-0.7%, 0.5%)
**Mental health medication use**						
Use of benzodiazepines; n (%)	12,216 (20.8%)	12,294 (20.9%)	-0.1% (-0.6%, 0.3%)	3,869 (22.3%)	3,700 (21.3%)	-1.0% (-1.8%, -0.1%)
Use of SNRIs; n (%)	3,591 (6.1%)	3,604 (6.1%)	-0.0% (-0.3%, 0.3%)	994 (5.7%)	964 (5.6%)	-0.2% (-0.7%, 0.3%)
Use of SSRIs; n (%)	10,177 (17.3%)	10,253 (17.5%)	-0.1% (-0.6%, 0.3%)	3,224 (18.6%)	3,050 (17.6%)	-1.0% (-1.8%, -0.2%)
Use of TCAs; n (%)	3,211 (5.5%)	3,242 (5.5%)	-0.1% (-0.3%, 0.2%)	875 (5.0%)	909 (5.2%)	0.2% (-0.3%, 0.7%)
Use of Non-BZD sedatives (Z-drugs); n (%)	4,624 (7.9%)	4,619 (7.9%)	0.0% (-0.3%, 0.3%)	1,369 (7.9%)	1,349 (7.8%)	-0.1% (-0.7%, 0.5%)
Use of atypical antipsychotics; n (%)	2,574 (4.4%)	2,746 (4.7%)	-0.3% (-0.5%, -0.1%)	756 (4.4%)	638 (3.7%)	-0.7% (-1.1%, -0.3%)
Use of typical antipsychotics; n (%)	298 (0.5%)	312 (0.5%)	-0.0% (-0.1%, 0.1%)	84 (0.5%)	87 (0.5%)	0.0% (-0.1%, 0.2%)
Use of anti-epileptics (other than mood stabilizers); n (%)	0 (0.0%)	0 (0.0%)	0.0% (0.0%, 0.0%)	0 (0.0%)	0 (0.0%)	0.0% (0.0%, 0.0%)
Use of anti-epileptic mood stabilizers; n (%)	0 (0.0%)	0 (0.0%)	0.0% (0.0%, 0.0%)	0 (0.0%)	0 (0.0%)	0.0% (0.0%, 0.0%)
**Cardiovascular diseases and risk factors**						
Obesity; n (%)	2,036 (3.5%)	2,040 (3.5%)	-0.0% (-0.2%, 0.2%)	689 (4.0%)	688 (4.0%)	-0.0% (-0.4%, 0.4%)
Smoking; n (%)	3,553 (6.1%)	3,692 (6.3%)	-0.2% (-0.5%, 0.0%)	1,484 (8.5%)	1,384 (8.0%)	-0.6% (-1.2%, 0.0%)
Hyperlipidemia; n (%)	10,730 (18.3%)	10,733 (18.3%)	-0.0% (-0.4%, 0.4%)	3,559 (20.5%)	3,551 (20.5%)	-0.0% (-0.9%, 0.8%)
Hypertension; n (%)	15,377 (26.2%)	15,602 (26.6%)	-0.4% (-0.9%, 0.1%)	4,584 (26.4%)	4,653 (26.8%)	0.4% (-0.5%, 1.3%)
Diabetes; n (%)	6,295 (10.7%)	6,317 (10.8%)	-0.0% (-0.4%, 0.3%)	1,660 (9.6%)	1,663 (9.6%)	0.0% (-0.6%, 0.6%)
VTE; n (%)	1,060 (1.8%)	1,070 (1.8%)	-0.0% (-0.2%, 0.1%)	350 (2.0%)	374 (2.2%)	0.1% (-0.2%, 0.4%)
Coronary revascularization (confounder); n (%)	0 (0.0%)	0 (0.0%)	0.0% (0.0%, 0.0%)	0 (0.0%)	0 (0.0%)	0.0% (0.0%, 0.0%)
Angina (stable and unstable); n (%)	722 (1.2%)	745 (1.3%)	-0.0% (-0.2%, 0.1%)	218 (1.3%)	221 (1.3%)	0.0% (-0.2%, 0.3%)
Myocardial infarction (as confounder); n (%)	0 (0.0%)	0 (0.0%)	0.0% (0.0%, 0.0%)	0 (0.0%)	0 (0.0%)	0.0% (0.0%, 0.0%)
Chronic kidney disease; n (%)	2,665 (4.5%)	2,666 (4.5%)	-0.0% (-0.2%, 0.2%)	753 (4.3%)	740 (4.3%)	-0.1% (-0.5%, 0.4%)
Other dysrhythmias; n (%)	2,838 (4.8%)	2,962 (5.0%)	-0.2% (-0.5%, 0.0%)	917 (5.3%)	979 (5.6%)	0.4% (-0.1%, 0.8%)
Stroke or TIA (Birman-Deych validation); n (%)	3,960 (6.7%)	3,903 (6.7%)	0.1% (-0.2%, 0.4%)	1,267 (7.3%)	1,346 (7.8%)	0.5% (-0.1%, 1.0%)
Atrial fibrillation; n (%)	1,659 (2.8%)	1,730 (2.9%)	-0.1% (-0.3%, 0.1%)	363 (2.1%)	374 (2.2%)	0.1% (-0.2%, 0.4%)
Valve disorders; n (%)	509 (0.9%)	525 (0.9%)	-0.0% (-0.1%, 0.1%)	195 (1.1%)	200 (1.2%)	0.0% (-0.2%, 0.3%)
Congestive heart failure; n (%)	450 (0.8%)	396 (0.7%)	0.1% (-0.0%, 0.2%)	85 (0.5%)	83 (0.5%)	-0.0% (-0.2%, 0.1%)
**Cardiovascular diseases and risk factor medication use**						
Use of insulin; n (%)	1,021 (1.7%)	1,039 (1.8%)	-0.0% (-0.2%, 0.1%)	251 (1.4%)	256 (1.5%)	0.0% (-0.2%, 0.3%)
Oral hypoglycemic medications; n (%)	4,202 (7.2%)	4,236 (7.2%)	-0.1% (-0.4%, 0.2%)	906 (5.2%)	894 (5.2%)	-0.1% (-0.5%, 0.4%)
Use of anti-arrhythmic drugs; n (%)	222 (0.4%)	235 (0.4%)	-0.0% (-0.1%, 0.1%)	157 (0.9%)	142 (0.8%)	-0.1% (-0.3%, 0.1%)
Use of beta blockers; n (%)	8,357 (14.2%)	8,453 (14.4%)	-0.2% (-0.6%, 0.2%)	1,827 (10.5%)	1,872 (10.8%)	0.3% (-0.4%, 0.9%)
Use of calcium channel blockers; n (%)	6,190 (10.5%)	6,183 (10.5%)	0.0% (-0.3%, 0.4%)	1,356 (7.8%)	1,393 (8.0%)	0.2% (-0.4%, 0.8%)
Use of ACE-I; n (%)	7,645 (13.0%)	7,677 (13.1%)	-0.1% (-0.4%, 0.3%)	1,743 (10.0%)	1,770 (10.2%)	0.2% (-0.5%, 0.8%)
Use of ARBs; n (%)	4,590 (7.8%)	4,613 (7.9%)	-0.0% (-0.3%, 0.3%)	863 (5.0%)	893 (5.1%)	0.2% (-0.3%, 0.6%)
Use of diuretics; n (%)	5,768 (9.8%)	5,820 (9.9%)	-0.1% (-0.4%, 0.3%)	1,195 (6.9%)	1,186 (6.8%)	-0.1% (-0.6%, 0.5%)
Use of other lipid-lowering drugs; n (%)	1,624 (2.8%)	1,656 (2.8%)	-0.1% (-0.2%, 0.1%)	351 (2.0%)	347 (2.0%)	-0.0% (-0.3%, 0.3%)
Use of statins; n (%)	11,513 (19.6%)	11,547 (19.7%)	-0.1% (-0.5%, 0.4%)	2,375 (13.7%)	2,391 (13.8%)	0.1% (-0.6%, 0.8%)
P2Y12 inhibitor use; n (%)	2,037 (3.5%)	1,983 (3.4%)	0.1% (-0.1%, 0.3%)	388 (2.2%)	399 (2.3%)	0.1% (-0.3%, 0.4%)
Use of aspirin; n (%)	662 (1.1%)	631 (1.1%)	0.1% (-0.1%, 0.2%)	227 (1.3%)	232 (1.3%)	0.0% (-0.2%, 0.3%)
Use of warfarin; n (%)	1,873 (3.2%)	1,857 (3.2%)	0.0% (-0.2%, 0.2%)	420 (2.4%)	440 (2.5%)	0.1% (-0.2%, 0.4%)
Use of NOACs; n (%)	122 (0.2%)	130 (0.2%)	-0.0% (-0.1%, 0.0%)	8 (0.0%)	10 (0.1%)	0.0% (-0.0%, 0.1%)
Use of heparin, LMWH, or fondaparinux; n (%)	1,099 (1.9%)	1,048 (1.8%)	0.1% (-0.1%, 0.2%)	191 (1.1%)	224 (1.3%)	0.2% (-0.0%, 0.4%)
Use of direct thrombin inhibitors; n (%)	6 (0.0%)	6 (0.0%)	0.0% (-0.0%, 0.0%)	0 (0.0%)	0 (0.0%)	0.0% (0.0%, 0.0%)
Use of glycoprotein IIb/IIIa inhibitors; n (%)	0 (0.0%)	0 (0.0%)	0.0% (0.0%, 0.0%)	0 (0.0%)	0 (0.0%)	0.0% (0.0%, 0.0%)
Use of tissue plasminogen activators; n (%)	32 (0.1%)	43 (0.1%)	-0.0% (-0.0%, 0.0%)	5 (0.0%)	7 (0.0%)	0.0% (-0.0%, 0.1%)
**Healthcare use characteristics**						
CV Hospitalizations; n (%)	1,413 (2.4%)	1,451 (2.5%)	-0.1% (-0.2%, 0.1%)	309 (1.8%)	391 (2.3%)	0.5% (0.2%, 0.8%)
Number of distinct medication prescriptions; mean (sd)	5.58 (5.10)	5.64 (5.14)	-0.05 (-0.11, 0.01)	5.22 (5.12)	5.13 (4.95)	-0.09 (-0.20, 0.01)
ED visits; n (%)	18,251 (31.1%)	19,063 (32.5%)	-1.4% (-1.9%, -0.8%)	6,116 (35.2%)	5,832 (33.6%)	-1.6% (-2.6%, -0.6%)

Supplementary Table 3. Patient characteristics, Mesalamine vs. Azathioprine/6-MP, 1:1 propensity score-matched.

	Truven			Optum		
**Variable**	**Mesalamine**	**Azathioprine/6-MP**	**Difference**	**Mesalamine**	**Azathioprine/6-MP**	**Difference**
**Number of patients**	20,464	20,464	-	6,841	6,841	-
**Demographics**						
Age; mean (sd)	42.37 (14.88)	42.88 (14.68)	0.51 (0.22, 0.80)	40.34 (13.10)	40.65 (13.41)	0.32 (-0.13, 0.76)
Female; n (%)	11,044 (54.0%)	11,182 (54.6%)	0.7% (-0.3%, 1.6%)	3,625 (53.0%)	3,684 (53.9%)	0.9% (-0.8%, 2.5%)
**Inflammatory bowel disease-related medication use and diagnoses**						
TNF inhibitors (IBD list); n (%)	4,344 (21.2%)	4,430 (21.6%)	0.4% (-0.4%, 1.2%)	1,072 (15.7%)	1,125 (16.4%)	0.8% (-0.5%, 2.0%)
Natalizumab; n (%)	9 (0.0%)	6 (0.0%)	-0.0% (-0.1%, 0.0%)	2 (0.0%)	2 (0.0%)	0.0% (-0.1%, 0.1%)
Non-biologics (IBD list); n (%)	450 (2.2%)	421 (2.1%)	-0.1% (-0.4%, 0.1%)	113 (1.7%)	102 (1.5%)	-0.2% (-0.6%, 0.3%)
ASA compounds; n (%)	2,465 (12.0%)	2,526 (12.3%)	0.3% (-0.3%, 0.9%)	1,038 (15.2%)	1,035 (15.1%)	-0.0% (-1.3%, 1.2%)
Systemic steroids; n (%)	8,790 (43.0%)	8,931 (43.6%)	0.7% (-0.3%, 1.7%)	3,328 (48.6%)	3,372 (49.3%)	0.6% (-1.0%, 2.3%)
Use of NSAIDs and Coxibs; n (%)	1,540 (7.5%)	1,620 (7.9%)	0.4% (-0.1%, 0.9%)	496 (7.3%)	555 (8.1%)	0.9% (-0.0%, 1.8%)
Intra-abdominal surgeries; n (%)	577 (2.8%)	612 (3.0%)	0.2% (-0.2%, 0.5%)	475 (6.9%)	529 (7.7%)	0.8% (-0.1%, 1.7%)
Weight loss; n (%)	323 (1.6%)	368 (1.8%)	0.2% (-0.0%, 0.5%)	137 (2.0%)	149 (2.2%)	0.2% (-0.3%, 0.7%)
Anemia; n (%)	1,564 (7.6%)	1,683 (8.2%)	0.6% (0.1%, 1.1%)	585 (8.6%)	624 (9.1%)	0.6% (-0.4%, 1.5%)
Fiztulizing or internal penetrating disease; n (%)	1,792 (8.8%)	1,941 (9.5%)	0.7% (0.2%, 1.3%)	771 (11.3%)	791 (11.6%)	0.3% (-0.8%, 1.4%)
Obstructing or stricturing disease; n (%)	1,838 (9.0%)	1,922 (9.4%)	0.4% (-0.2%, 1.0%)	772 (11.3%)	762 (11.1%)	-0.1% (-1.2%, 0.9%)
Volume depletion; n (%)	1,490 (7.3%)	1,599 (7.8%)	0.5% (0.0%, 1.0%)	576 (8.4%)	630 (9.2%)	0.8% (-0.2%, 1.8%)
Cdiff infections; n (%)	213 (1.0%)	225 (1.1%)	0.1% (-0.1%, 0.3%)	61 (0.9%)	76 (1.1%)	0.2% (-0.1%, 0.6%)
**Cardiovascular diseases and risk factors**						
Coronary revascularization (confounder); n (%)	0 (0.0%)	0 (0.0%)	0.0% (0.0%, 0.0%)	0 (0.0%)	0 (0.0%)	0.0% (0.0%, 0.0%)
Angina (stable and unstable); n (%)	104 (0.5%)	98 (0.5%)	-0.0% (-0.2%, 0.1%)	48 (0.7%)	37 (0.5%)	-0.2% (-0.4%, 0.1%)
Myocardial infarction (as confounder); n (%)	0 (0.0%)	0 (0.0%)	0.0% (0.0%, 0.0%)	0 (0.0%)	0 (0.0%)	0.0% (0.0%, 0.0%)
Atrial fibrillation; n (%)	241 (1.2%)	238 (1.2%)	-0.0% (-0.2%, 0.2%)	45 (0.7%)	51 (0.7%)	0.1% (-0.2%, 0.4%)
Other dysrhythmias; n (%)	525 (2.6%)	581 (2.8%)	0.3% (-0.0%, 0.6%)	156 (2.3%)	191 (2.8%)	0.5% (-0.0%, 1.1%)
Valve disorders; n (%)	75 (0.4%)	91 (0.4%)	0.1% (-0.0%, 0.2%)	35 (0.5%)	40 (0.6%)	0.1% (-0.2%, 0.3%)
Congestive heart failure; n (%)	58 (0.3%)	65 (0.3%)	0.0% (-0.1%, 0.1%)	10 (0.1%)	10 (0.1%)	0.0% (-0.1%, 0.1%)
Stroke or TIA (Birman-Deych validation); n (%)	137 (0.7%)	147 (0.7%)	0.0% (-0.1%, 0.2%)	51 (0.7%)	58 (0.8%)	0.1% (-0.2%, 0.4%)
Chronic kidney disease; n (%)	926 (4.5%)	941 (4.6%)	0.1% (-0.3%, 0.5%)	329 (4.8%)	343 (5.0%)	0.2% (-0.5%, 0.9%)
Diabetes; n (%)	1,163 (5.7%)	1,245 (6.1%)	0.4% (-0.1%, 0.9%)	337 (4.9%)	344 (5.0%)	0.1% (-0.6%, 0.8%)
Hyperlipidemia; n (%)	2,169 (10.6%)	2,342 (11.4%)	0.8% (0.2%, 1.5%)	690 (10.1%)	747 (10.9%)	0.8% (-0.2%, 1.9%)
Hypertension; n (%)	2,904 (14.2%)	3,125 (15.3%)	1.1% (0.4%, 1.8%)	914 (13.4%)	996 (14.6%)	1.2% (0.0%, 2.4%)
Smoking; n (%)	1,007 (4.9%)	1,044 (5.1%)	0.2% (-0.2%, 0.6%)	391 (5.7%)	435 (6.4%)	0.6% (-0.2%, 1.5%)
Obesity; n (%)	561 (2.7%)	584 (2.9%)	0.1% (-0.2%, 0.4%)	146 (2.1%)	173 (2.5%)	0.4% (-0.1%, 0.9%)
**Medication use for cardiovascular diseases and risk factors**						
Use of insulin; n (%)	215 (1.1%)	208 (1.0%)	-0.0% (-0.2%, 0.2%)	63 (0.9%)	63 (0.9%)	0.0% (-0.3%, 0.3%)
Oral hypoglycemic medications; n (%)	630 (3.1%)	694 (3.4%)	0.3% (-0.0%, 0.7%)	179 (2.6%)	184 (2.7%)	0.1% (-0.5%, 0.6%)
Use of beta blockers; n (%)	1,501 (7.3%)	1,545 (7.5%)	0.2% (-0.3%, 0.7%)	331 (4.8%)	375 (5.5%)	0.6% (-0.1%, 1.4%)
Use of calcium channel blockers; n (%)	1,002 (4.9%)	1,067 (5.2%)	0.3% (-0.1%, 0.7%)	250 (3.7%)	259 (3.8%)	0.1% (-0.5%, 0.8%)
Use of ARBs; n (%)	807 (3.9%)	866 (4.2%)	0.3% (-0.1%, 0.7%)	158 (2.3%)	187 (2.7%)	0.4% (-0.1%, 1.0%)
Use of ACE-I; n (%)	1,338 (6.5%)	1,441 (7.0%)	0.5% (0.0%, 1.0%)	350 (5.1%)	391 (5.7%)	0.6% (-0.2%, 1.4%)
Use of anti-arrhythmic drugs; n (%)	103 (0.5%)	105 (0.5%)	0.0% (-0.1%, 0.2%)	68 (1.0%)	73 (1.1%)	0.1% (-0.3%, 0.4%)
Use of statins; n (%)	1,449 (7.1%)	1,598 (7.8%)	0.7% (0.2%, 1.2%)	328 (4.8%)	359 (5.2%)	0.5% (-0.3%, 1.2%)
Use of other lipid-lowering drugs; n (%)	556 (2.7%)	591 (2.9%)	0.2% (-0.2%, 0.5%)	134 (2.0%)	156 (2.3%)	0.3% (-0.2%, 0.8%)
Use of aspirin; n (%)	60 (0.3%)	67 (0.3%)	0.0% (-0.1%, 0.1%)	16 (0.2%)	22 (0.3%)	0.1% (-0.1%, 0.3%)
Use of diuretics; n (%)	1,006 (4.9%)	1,081 (5.3%)	0.4% (-0.1%, 0.8%)	293 (4.3%)	308 (4.5%)	0.2% (-0.5%, 0.9%)
Use of warfarin; n (%)	340 (1.7%)	358 (1.7%)	0.1% (-0.2%, 0.3%)	89 (1.3%)	92 (1.3%)	0.0% (-0.4%, 0.4%)
P2Y12 inhibitor use; n (%)	125 (0.6%)	151 (0.7%)	0.1% (-0.0%, 0.3%)	39 (0.6%)	37 (0.5%)	-0.0% (-0.3%, 0.2%)
Use of glycoprotein IIb/IIIa inhibitors; n (%)	0 (0.0%)	0 (0.0%)	0.0% (0.0%, 0.0%)	0 (0.0%)	0 (0.0%)	0.0% (0.0%, 0.0%)
Use of direct thrombin inhibitors; n (%)	0 (0.0%)	0 (0.0%)	0.0% (0.0%, 0.0%)	0 (0.0%)	0 (0.0%)	0.0% (0.0%, 0.0%)
Use of tissue plasminogen activators; n (%)	28 (0.1%)	29 (0.1%)	0.0% (-0.1%, 0.1%)	8 (0.1%)	6 (0.1%)	-0.0% (-0.2%, 0.1%)
Use of heparin, LMWH, or fondaparinux; n (%)	344 (1.7%)	339 (1.7%)	-0.0% (-0.3%, 0.2%)	74 (1.1%)	72 (1.1%)	-0.0% (-0.4%, 0.3%)
**Healthcare use characteristics**						
ED visits; n (%)	4,907 (24.0%)	5,200 (25.4%)	1.4% (0.6%, 2.3%)	1,583 (23.1%)	1,756 (25.7%)	2.5% (1.1%, 4.0%)
Number of distinct medication prescriptions; mean (sd)	5.26 (4.68)	5.47 (4.89)	0.21 (0.11, 0.30)	5.00 (4.48)	5.28 (4.68)	0.28 (0.13, 0.43)
CV Hospitalizations; n (%)	152 (0.7%)	158 (0.8%)	0.0% (-0.1%, 0.2%)	46 (0.7%)	44 (0.6%)	-0.0% (-0.3%, 0.3%)

Supplementary Table 4. Patient characteristics, Lithium vs. Lamotrigine, 1:1 propensity score-matched.

	Truven			Optum		
**Variable**	**Lithium exposure**	**Lamotrigine exposure**	**Difference**	**Lithium exposure**	**Lamotrigine exposure**	**Difference**
**Number of patients**	99,656	99,656	-	41,638	41,638	-
**Demographics**						
Age; mean (sd)	42.17 (14.53)	42.34 (14.46)	0.17 (0.05, 0.30)	39.94 (12.74)	40.02 (12.61)	0.07 (-0.10, 0.24)
Female; n (%)	57,889 (58.1%)	57,336 (57.5%)	-0.6% (-1.0%, -0.1%)	24,302 (58.4%)	24,241 (58.2%)	-0.1% (-0.8%, 0.5%)
**Mental health diagnoses**						
Epilepsy; n (%)	2,079 (2.1%)	1,800 (1.8%)	-0.3% (-0.4%, -0.2%)	922 (2.2%)	859 (2.1%)	-0.2% (-0.4%, 0.0%)
Anxiety; n (%)	10,006 (10.0%)	9,794 (9.8%)	-0.2% (-0.5%, 0.1%)	4,910 (11.8%)	4,740 (11.4%)	-0.4% (-0.8%, 0.0%)
Depression; n (%)	10,514 (10.6%)	10,213 (10.2%)	-0.3% (-0.6%, -0.0%)	5,432 (13.0%)	5,285 (12.7%)	-0.4% (-0.8%, 0.1%)
Sleep disorders; n (%)	8,872 (8.9%)	8,533 (8.6%)	-0.3% (-0.6%, -0.1%)	4,409 (10.6%)	4,178 (10.0%)	-0.6% (-1.0%, -0.1%)
Bipolar disorder; n (%)	2,526 (2.5%)	2,209 (2.2%)	-0.3% (-0.5%, -0.2%)	969 (2.3%)	894 (2.1%)	-0.2% (-0.4%, 0.0%)
Drug or alcohol abuse; n (%)	12,521 (12.6%)	12,232 (12.3%)	-0.3% (-0.6%, 0.0%)	5,906 (14.2%)	5,816 (14.0%)	-0.2% (-0.7%, 0.3%)
Psychotic disorders; n (%)	9,209 (9.2%)	8,460 (8.5%)	-0.8% (-1.0%, -0.5%)	3,186 (7.7%)	2,903 (7.0%)	-0.7% (-1.0%, -0.3%)
Neuropathic pain; n (%)	8,347 (8.4%)	8,229 (8.3%)	-0.1% (-0.4%, 0.1%)	3,660 (8.8%)	3,396 (8.2%)	-0.6% (-1.0%, -0.3%)
Migraine; n (%)	5,992 (6.0%)	5,681 (5.7%)	-0.3% (-0.5%, -0.1%)	2,919 (7.0%)	2,709 (6.5%)	-0.5% (-0.8%, -0.2%)
**Mental health medication use**						
Use of benzodiazepines; n (%)	40,690 (40.8%)	40,368 (40.5%)	-0.3% (-0.8%, 0.1%)	16,225 (39.0%)	15,852 (38.1%)	-0.9% (-1.6%, -0.2%)
Use of SNRIs; n (%)	18,591 (18.7%)	18,161 (18.2%)	-0.4% (-0.8%, -0.1%)	7,118 (17.1%)	6,917 (16.6%)	-0.5% (-1.0%, 0.0%)
Use of SSRIs; n (%)	36,473 (36.6%)	36,083 (36.2%)	-0.4% (-0.8%, 0.0%)	15,650 (37.6%)	15,430 (37.1%)	-0.5% (-1.2%, 0.1%)
Use of TCAs; n (%)	6,488 (6.5%)	6,271 (6.3%)	-0.2% (-0.4%, -0.0%)	2,499 (6.0%)	2,432 (5.8%)	-0.2% (-0.5%, 0.2%)
Use of Non-BZD sedatives (Z-drugs); n (%)	15,134 (15.2%)	14,758 (14.8%)	-0.4% (-0.7%, -0.1%)	5,856 (14.1%)	5,664 (13.6%)	-0.5% (-0.9%, 0.0%)
Use of atypical antipsychotics; n (%)	29,767 (29.9%)	29,623 (29.7%)	-0.1% (-0.5%, 0.3%)	10,769 (25.9%)	10,491 (25.2%)	-0.7% (-1.3%, -0.1%)
Use of typical antipsychotics; n (%)	2,021 (2.0%)	1,794 (1.8%)	-0.2% (-0.3%, -0.1%)	666 (1.6%)	599 (1.4%)	-0.2% (-0.3%, 0.0%)
Use of anti-epileptics (other than mood stabilizers); n (%)	13,247 (13.3%)	12,764 (12.8%)	-0.5% (-0.8%, -0.2%)	5,333 (12.8%)	5,011 (12.0%)	-0.8% (-1.2%, -0.3%)
Use of anti-epileptic mood stabilizers; n (%)	13,708 (13.8%)	13,400 (13.4%)	-0.3% (-0.6%, -0.0%)	6,145 (14.8%)	5,977 (14.4%)	-0.4% (-0.9%, 0.1%)
**Cardiovascular diseases and risk factors**						
Obesity; n (%)	4,319 (4.3%)	4,070 (4.1%)	-0.2% (-0.4%, -0.1%)	1,768 (4.2%)	1,685 (4.0%)	-0.2% (-0.5%, 0.1%)
Smoking; n (%)	8,159 (8.2%)	7,881 (7.9%)	-0.3% (-0.5%, -0.0%)	3,877 (9.3%)	3,738 (9.0%)	-0.3% (-0.7%, 0.1%)
Hyperlipidemia; n (%)	14,459 (14.5%)	14,158 (14.2%)	-0.3% (-0.6%, 0.0%)	6,071 (14.6%)	5,773 (13.9%)	-0.7% (-1.2%, -0.2%)
Hypertension; n (%)	16,827 (16.9%)	16,280 (16.3%)	-0.5% (-0.9%, -0.2%)	6,485 (15.6%)	6,259 (15.0%)	-0.5% (-1.0%, -0.1%)
Diabetes; n (%)	7,395 (7.4%)	7,109 (7.1%)	-0.3% (-0.5%, -0.1%)	2,470 (5.9%)	2,371 (5.7%)	-0.2% (-0.6%, 0.1%)
VTE; n (%)	824 (0.8%)	765 (0.8%)	-0.1% (-0.1%, 0.0%)	309 (0.7%)	303 (0.7%)	-0.0% (-0.1%, 0.1%)
Coronary revascularization (confounder); n (%)	148 (0.1%)	144 (0.1%)	-0.0% (-0.0%, 0.0%)	43 (0.1%)	38 (0.1%)	-0.0% (-0.1%, 0.0%)
Angina (stable and unstable); n (%)	732 (0.7%)	716 (0.7%)	-0.0% (-0.1%, 0.1%)	350 (0.8%)	323 (0.8%)	-0.1% (-0.2%, 0.1%)
Myocardial infarction (as confounder); n (%)	213 (0.2%)	222 (0.2%)	0.0% (-0.0%, 0.1%)	89 (0.2%)	94 (0.2%)	0.0% (-0.1%, 0.1%)
Congestive heart failure; n (%)	180 (0.2%)	189 (0.2%)	0.0% (-0.0%, 0.0%)	46 (0.1%)	35 (0.1%)	-0.0% (-0.1%, 0.0%)
Chronic kidney disease; n (%)	2,060 (2.1%)	1,993 (2.0%)	-0.1% (-0.2%, 0.1%)	707 (1.7%)	672 (1.6%)	-0.1% (-0.3%, 0.1%)
Other dysrhythmias; n (%)	3,065 (3.1%)	2,941 (3.0%)	-0.1% (-0.3%, 0.0%)	1,124 (2.7%)	1,085 (2.6%)	-0.1% (-0.3%, 0.1%)
Atrial fibrillation; n (%)	706 (0.7%)	698 (0.7%)	-0.0% (-0.1%, 0.1%)	201 (0.5%)	178 (0.4%)	-0.1% (-0.1%, 0.0%)
Valve disorders; n (%)	316 (0.3%)	292 (0.3%)	-0.0% (-0.1%, 0.0%)	155 (0.4%)	140 (0.3%)	-0.0% (-0.1%, 0.0%)
Stroke or TIA (Birman-Deych validation); n (%)	0 (0.0%)	0 (0.0%)	0.0% (0.0%, 0.0%)	0 (0.0%)	0 (0.0%)	0.0% (0.0%, 0.0%)
**Medication use for cardiovascular diseases and risk factors**						
Use of insulin; n (%)	1,143 (1.1%)	1,129 (1.1%)	-0.0% (-0.1%, 0.1%)	358 (0.9%)	368 (0.9%)	0.0% (-0.1%, 0.2%)
Oral hypoglycemic medications; n (%)	5,233 (5.3%)	5,088 (5.1%)	-0.1% (-0.3%, 0.1%)	1,587 (3.8%)	1,494 (3.6%)	-0.2% (-0.5%, 0.0%)
Use of anti-arrhythmic drugs; n (%)	232 (0.2%)	199 (0.2%)	-0.0% (-0.1%, 0.0%)	259 (0.6%)	265 (0.6%)	0.0% (-0.1%, 0.1%)
Use of beta blockers; n (%)	9,460 (9.5%)	9,176 (9.2%)	-0.3% (-0.5%, -0.0%)	2,965 (7.1%)	2,874 (6.9%)	-0.2% (-0.6%, 0.1%)
Use of calcium channel blockers; n (%)	5,813 (5.8%)	5,713 (5.7%)	-0.1% (-0.3%, 0.1%)	1,855 (4.5%)	1,813 (4.4%)	-0.1% (-0.4%, 0.2%)
Use of ACE-I; n (%)	8,163 (8.2%)	7,846 (7.9%)	-0.3% (-0.6%, -0.1%)	2,545 (6.1%)	2,450 (5.9%)	-0.2% (-0.6%, 0.1%)
Use of ARBs; n (%)	4,150 (4.2%)	3,994 (4.0%)	-0.2% (-0.3%, 0.0%)	1,158 (2.8%)	1,114 (2.7%)	-0.1% (-0.3%, 0.1%)
Use of diuretics; n (%)	5,528 (5.5%)	5,316 (5.3%)	-0.2% (-0.4%, -0.0%)	1,628 (3.9%)	1,554 (3.7%)	-0.2% (-0.4%, 0.1%)
Use of other lipid-lowering drugs; n (%)	2,001 (2.0%)	1,901 (1.9%)	-0.1% (-0.2%, 0.0%)	565 (1.4%)	541 (1.3%)	-0.1% (-0.2%, 0.1%)
Use of statins; n (%)	12,098 (12.1%)	11,957 (12.0%)	-0.1% (-0.4%, 0.1%)	3,471 (8.3%)	3,363 (8.1%)	-0.3% (-0.6%, 0.1%)
P2Y12 inhibitor use; n (%)	958 (1.0%)	956 (1.0%)	-0.0% (-0.1%, 0.1%)	199 (0.5%)	211 (0.5%)	0.0% (-0.1%, 0.1%)
Use of aspirin; n (%)	697 (0.7%)	713 (0.7%)	0.0% (-0.1%, 0.1%)	433 (1.0%)	420 (1.0%)	-0.0% (-0.2%, 0.1%)
Use of warfarin; n (%)	943 (0.9%)	922 (0.9%)	-0.0% (-0.1%, 0.1%)	285 (0.7%)	263 (0.6%)	-0.1% (-0.2%, 0.1%)
Use of NOACs; n (%)	73 (0.1%)	72 (0.1%)	-0.0% (-0.0%, 0.0%)	8 (0.0%)	6 (0.0%)	-0.0% (-0.0%, 0.0%)
Use of heparin, LMWH, or fondaparinux; n (%)	805 (0.8%)	783 (0.8%)	-0.0% (-0.1%, 0.1%)	156 (0.4%)	141 (0.3%)	-0.0% (-0.1%, 0.0%)
Use of direct thrombin inhibitors; n (%)	8 (0.0%)	7 (0.0%)	-0.0% (-0.0%, 0.0%)	0 (0.0%)	0 (0.0%)	0.0% (0.0%, 0.0%)
Use of glycoprotein IIb/IIIa inhibitors; n (%)	1 (0.0%)	2 (0.0%)	0.0% (-0.0%, 0.0%)	0 (0.0%)	0 (0.0%)	0.0% (0.0%, 0.0%)
Use of tissue plasminogen activators; n (%)	22 (0.0%)	22 (0.0%)	0.0% (-0.0%, 0.0%)	4 (0.0%)	6 (0.0%)	0.0% (-0.0%, 0.0%)
**Healthcare use characteristics**						
CV Hospitalizations; n (%)	538 (0.5%)	534 (0.5%)	-0.0% (-0.1%, 0.1%)	132 (0.3%)	132 (0.3%)	0.0% (-0.1%, 0.1%)
Number of distinct medication prescriptions; mean (sd)	6.61 (5.47)	6.50 (5.27)	-0.11 (-0.16, -0.06)	6.17 (5.30)	6.01 (4.97)	-0.16 (-0.23, -0.09)
ED visits; n (%)	30,342 (30.4%)	29,535 (29.6%)	-0.8% (-1.2%, -0.4%)	11,447 (27.5%)	11,064 (26.6%)	-0.9% (-1.5%, -0.3%)

Supplementary Table 5. Patient characteristics, Hydroxychloroquine (HCQ) vs. Leflunomide, 1:1 propensity score-matched.

	Truven			Optum		
**Variable**	**HCQ exposure**	**Leflunomide exposure**	**Difference**	**HCQ exposure**	**Leflunomide exposure**	**Difference**
**Number of patients**	29,080	29,080	-	8,715	8,715	-
**Demographics**						
Age; mean (sd)	57.22 (13.27)	56.37 (12.57)	-0.85 (-1.06, -0.64)	52.39 (12.03)	51.91 (11.31)	-0.47 (-0.82, -0.13)
Female; n (%)	22,195 (76.3%)	22,432 (77.1%)	0.8% (0.1%, 1.5%)	6,586 (75.6%)	6,623 (76.0%)	0.4% (-0.9%, 1.7%)
**Cardiovascular diseases and risk factors**						
Obesity; n (%)	1,134 (3.9%)	1,183 (4.1%)	0.2% (-0.2%, 0.5%)	327 (3.8%)	353 (4.1%)	0.3% (-0.3%, 0.9%)
Smoking; n (%)	1,220 (4.2%)	1,182 (4.1%)	-0.1% (-0.5%, 0.2%)	387 (4.4%)	434 (5.0%)	0.5% (-0.1%, 1.2%)
Hypertension; n (%)	9,475 (32.6%)	9,294 (32.0%)	-0.6% (-1.4%, 0.1%)	2,585 (29.7%)	2,672 (30.7%)	1.0% (-0.4%, 2.4%)
Hyperlipidemia; n (%)	6,210 (21.4%)	6,226 (21.4%)	0.1% (-0.6%, 0.7%)	1,936 (22.2%)	2,054 (23.6%)	1.4% (0.1%, 2.6%)
Diabetes; n (%)	4,096 (14.1%)	3,985 (13.7%)	-0.4% (-0.9%, 0.2%)	991 (11.4%)	1,030 (11.8%)	0.4% (-0.5%, 1.4%)
VTE; n (%)	645 (2.2%)	628 (2.2%)	-0.1% (-0.3%, 0.2%)	181 (2.1%)	187 (2.1%)	0.1% (-0.4%, 0.5%)
Coronary revascularization (confounder); n (%)	0 (0.0%)	0 (0.0%)	0.0% (0.0%, 0.0%)	0 (0.0%)	0 (0.0%)	0.0% (0.0%, 0.0%)
Angina (stable and unstable); n (%)	429 (1.5%)	440 (1.5%)	0.0% (-0.2%, 0.2%)	85 (1.0%)	104 (1.2%)	0.2% (-0.1%, 0.5%)
Myocardial infarction (as confounder); n (%)	0 (0.0%)	0 (0.0%)	0.0% (0.0%, 0.0%)	0 (0.0%)	0 (0.0%)	0.0% (0.0%, 0.0%)
Chronic kidney disease; n (%)	1,638 (5.6%)	1,544 (5.3%)	-0.3% (-0.7%, 0.0%)	343 (3.9%)	390 (4.5%)	0.5% (-0.1%, 1.1%)
Other dysrhythmias; n (%)	1,077 (3.7%)	1,061 (3.6%)	-0.1% (-0.4%, 0.3%)	258 (3.0%)	256 (2.9%)	-0.0% (-0.5%, 0.5%)
Atrial fibrillation; n (%)	822 (2.8%)	796 (2.7%)	-0.1% (-0.4%, 0.2%)	157 (1.8%)	153 (1.8%)	-0.0% (-0.4%, 0.4%)
Valve disorders; n (%)	229 (0.8%)	229 (0.8%)	0.0% (-0.1%, 0.1%)	82 (0.9%)	75 (0.9%)	-0.1% (-0.4%, 0.2%)
Congestive heart failure; n (%)	255 (0.9%)	256 (0.9%)	0.0% (-0.2%, 0.2%)	44 (0.5%)	45 (0.5%)	0.0% (-0.2%, 0.2%)
Stroke or TIA (Birman-Deych validation); n (%)	490 (1.7%)	460 (1.6%)	-0.1% (-0.3%, 0.1%)	116 (1.3%)	116 (1.3%)	0.0% (-0.3%, 0.3%)
**Medication use for cardiovascular diseases and risk factors**						
Use of insulin; n (%)	745 (2.6%)	726 (2.5%)	-0.1% (-0.3%, 0.2%)	158 (1.8%)	170 (2.0%)	0.1% (-0.3%, 0.6%)
Oral hypoglycemic medications; n (%)	2,712 (9.3%)	2,689 (9.2%)	-0.1% (-0.6%, 0.4%)	604 (6.9%)	631 (7.2%)	0.3% (-0.5%, 1.1%)
Use of anti-arrhythmic drugs; n (%)	147 (0.5%)	151 (0.5%)	0.0% (-0.1%, 0.1%)	89 (1.0%)	98 (1.1%)	0.1% (-0.2%, 0.4%)
Use of beta blockers; n (%)	5,203 (17.9%)	5,064 (17.4%)	-0.5% (-1.1%, 0.1%)	1,048 (12.0%)	1,076 (12.3%)	0.3% (-0.7%, 1.3%)
Use of calcium channel blockers; n (%)	4,256 (14.6%)	4,169 (14.3%)	-0.3% (-0.9%, 0.3%)	874 (10.0%)	911 (10.5%)	0.4% (-0.5%, 1.3%)
Use of ACE-I; n (%)	5,071 (17.4%)	4,913 (16.9%)	-0.5% (-1.2%, 0.1%)	1,116 (12.8%)	1,122 (12.9%)	0.1% (-0.9%, 1.1%)
Use of ARBs; n (%)	3,594 (12.4%)	3,574 (12.3%)	-0.1% (-0.6%, 0.5%)	705 (8.1%)	750 (8.6%)	0.5% (-0.3%, 1.3%)
Use of diuretics; n (%)	4,796 (16.5%)	4,701 (16.2%)	-0.3% (-0.9%, 0.3%)	995 (11.4%)	1,023 (11.7%)	0.3% (-0.6%, 1.3%)
Use of other lipid-lowering drugs; n (%)	999 (3.4%)	1,027 (3.5%)	0.1% (-0.2%, 0.4%)	192 (2.2%)	215 (2.5%)	0.3% (-0.2%, 0.7%)
P2Y12 inhibitor use; n (%)	972 (3.3%)	911 (3.1%)	-0.2% (-0.5%, 0.1%)	125 (1.4%)	143 (1.6%)	0.2% (-0.2%, 0.6%)
Use of statins; n (%)	6,769 (23.3%)	6,648 (22.9%)	-0.4% (-1.1%, 0.3%)	1,322 (15.2%)	1,378 (15.8%)	0.6% (-0.4%, 1.7%)
Use of aspirin; n (%)	193 (0.7%)	196 (0.7%)	0.0% (-0.1%, 0.1%)	90 (1.0%)	90 (1.0%)	0.0% (-0.3%, 0.3%)
Use of warfarin; n (%)	1,101 (3.8%)	1,036 (3.6%)	-0.2% (-0.5%, 0.1%)	250 (2.9%)	245 (2.8%)	-0.1% (-0.6%, 0.4%)
Use of NOACs; n (%)	134 (0.5%)	124 (0.4%)	-0.0% (-0.1%, 0.1%)	8 (0.1%)	11 (0.1%)	0.0% (-0.1%, 0.1%)
Use of heparin, LMWH, or fondaparinux; n (%)	543 (1.9%)	520 (1.8%)	-0.1% (-0.3%, 0.1%)	119 (1.4%)	118 (1.4%)	-0.0% (-0.4%, 0.3%)
Use of direct thrombin inhibitors; n (%)	8 (0.0%)	6 (0.0%)	-0.0% (-0.0%, 0.0%)	0 (0.0%)	0 (0.0%)	0.0% (0.0%, 0.0%)
Use of glycoprotein IIb/IIIa inhibitors; n (%)	1 (0.0%)	1 (0.0%)	0.0% (-0.0%, 0.0%)	0 (0.0%)	0 (0.0%)	0.0% (0.0%, 0.0%)
Use of tissue plasminogen activators; n (%)	25 (0.1%)	24 (0.1%)	-0.0% (-0.1%, 0.0%)	6 (0.1%)	5 (0.1%)	-0.0% (-0.1%, 0.1%)
**Rheumatoid arthritis related medication use**						
MTX exposure; n (%)	13,683 (47.1%)	12,898 (44.4%)	-2.7% (-3.5%, -1.9%)	4,343 (49.8%)	3,986 (45.7%)	-4.1% (-5.6%, -2.6%)
SSZ exposure; n (%)	2,058 (7.1%)	1,897 (6.5%)	-0.6% (-1.0%, -0.1%)	640 (7.3%)	595 (6.8%)	-0.5% (-1.3%, 0.3%)
Other non-biologics- RA list; n (%)	1,355 (4.7%)	1,392 (4.8%)	0.1% (-0.2%, 0.5%)	371 (4.3%)	406 (4.7%)	0.4% (-0.2%, 1.0%)
TNF inhibitors (RA list); n (%)	7,176 (24.7%)	7,888 (27.1%)	2.4% (1.7%, 3.2%)	2,013 (23.1%)	2,298 (26.4%)	3.3% (2.0%, 4.6%)
nonTNF biologics (RA list); n (%)	1,414 (4.9%)	1,672 (5.7%)	0.9% (0.5%, 1.3%)	256 (2.9%)	333 (3.8%)	0.9% (0.3%, 1.4%)
Use of NSAIDs and Coxibs; n (%)	10,457 (36.0%)	10,698 (36.8%)	0.8% (0.0%, 1.6%)	3,481 (39.9%)	3,620 (41.5%)	1.6% (0.1%, 3.1%)
Systemic steroids; n (%)	16,397 (56.4%)	16,345 (56.2%)	-0.2% (-1.0%, 0.6%)	5,767 (66.2%)	5,842 (67.0%)	0.9% (-0.6%, 2.3%)
**Healthcare use characteristics**						
CV Hospitalizations; n (%)	423 (1.5%)	390 (1.3%)	-0.1% (-0.3%, 0.1%)	69 (0.8%)	64 (0.7%)	-0.1% (-0.3%, 0.2%)
Number of distinct medication prescriptions; mean (sd)	8.93 (5.61)	8.97 (5.68)	0.04 (-0.05, 0.13)	7.87 (5.32)	8.14 (5.44)	0.28 (0.12, 0.44)
ED visits; n (%)	5,028 (17.3%)	5,085 (17.5%)	0.2% (-0.4%, 0.8%)	1,197 (13.7%)	1,298 (14.9%)	1.2% (0.1%, 2.2%)

Supplementary References

1 Law, V. *et al.* DrugBank 4.0: shedding new light on drug metabolism. *Nucleic Acids Res.* **42**, D1091-1097 (2014).

2 Ursu, O. *et al.* DrugCentral: online drug compendium. *Nucleic Acids Res.* **45**, D932-D939 (2017).

3 Wei, W. Q. *et al.* Development and evaluation of an ensemble resource linking medications to their indications. *J. Am. Med. Inform. Assoc.* **20**, 954-961 (2013).

4 Guney, E., Menche, J., Vidal, M. & Barabasi, A. L. Network-based in silico drug efficacy screening. *Nat. Commun.* **7**, 10331 (2016).
