# Supplementary material for: Network-based approach to prediction and population-based validation of in silico drug repurposing
Source: Nat Commun. 2018 Jul 12;9:2691. doi: 10.1038/s41467-018-05116-5 (PMC6043492; doi:10.1038/s41467-018-05116-5)
Supplement: Supplementary file 3 — Description of Additional Supplementary Files [file 41467_2018_5116_MOESM3_ESM.pdf]

Description of Additional Supplementary Files

File Name: Supplementary Data 1
Description: The updated human interactome contains 217,160 publicly available protein-protein interactions. (.tsv)

File Name: Supplementary Data 2
Description: The global predicted z-scores for 984 FDA-approved drugs and 23 types of cardiovascular events (diseases) via the network proximity approach. (.xlsx)
